# Antioxidant Diets and Lifestyles Could Mitigate the Risk of Sarcopenia with Low Muscle Mass in Women: A Retrospective Study

**DOI:** 10.3390/healthcare13080910

**Published:** 2025-04-15

**Authors:** Shanshan Li, Yiqiong Zhang, Qian Li, Wenjun Liu, Yue Wu

**Affiliations:** 1 Department of Cardiovascular Medicine, The First Affiliated Hospital, Xi’an Jiaotong University, Xi’an 710061, China; ls499261982@stu.xjtu.edu.cn (S.L.); 4120115045@stu.xjtu.edu.cn (Y.Z.); 4120315008@stu.xjtu.edu.cn (Q.L.); 2 Key Laboratory of Molecular Cardiology, Xi’an 710061, China; 3 Key Laboratory of Environment and Genes Related to Diseases, Ministry of Education, Xi’an 710061, China

**Keywords:** sarcopenia, oxidative balance score, antioxidant, physical activity, diet nutrition

## Abstract

**Background:** Sarcopenia is characterized by a loss of muscle mass, strength, and function. At present, there are no effective methods available for prevention or treatment. Oxidative Balance Score (OBS) has been shown to be significantly correlated with a decreased risk of sarcopenia. Nevertheless, gender-specific studies still exhibit certain limitations. **Methods:** Individuals who completed dual-energy X-ray absorptiometry and the diet and lifestyle questionnaires from the National Health and Nutrition Examination Survey were enrolled. OBSs were calculated according to dietary or lifestyle variables and physical activity. Sarcopenia with low muscle mass (SLM) was identified based on the ratio of appendicular skeletal muscle mass to body mass index. A logistic regression analysis investigated the association between OBS and the risk of SLM in different gender groups. Kaplan–Meier survival and subgroup analyses and Cox regression analyses were used to explore the relationship between OBS and mortality in individuals with SLM in each gender subgroup. **Results:** The level of OBS in the SLM group was lower (20.40 vs. 17.07, *p* < 0.05). A multivariate logistic regression analysis showed that the OR between individual dietary nutrients and lifestyle and the risk of SLM exhibition was gender-specific. Stratified analyses revealed that total OBS, as well as diet and lifestyle OBS were negatively associated with the risk of SLM within each gender subgroup (all *p* < 0.05, all trends *p* < 0.05). Furthermore, a restriction cubic spline regression analysis showed that diet and lifestyle OBSs were negatively correlated with the risk of SLM in females (nonlinear *p* = 0.0469, nonlinear *p =* 0.0254). The KM curve showed that OBS was not significantly associated with all-cause mortality in the male and female subgroups (female, *p =* 0.064, male, *p =* 0.53). **Conclusions:** This study establishes a significant negative correlation between OBS and the risk of SLM, particularly among females. Consequently, adopting an antioxidant diet and lifestyle may prove to be more advantageous for females.

## 1. Introduction

Sarcopenia is an age-related, progressive disease characterized by a loss of muscle mass, strength, and function that mainly affects the elderly but now tends to be affecting younger people as well [1]. Due to the varying classifications and cutoffs for skeletal muscle mass and function, the prevalence of sarcopenia ranges from 10 to 27% [2]. Moreover, there is evidence linking sarcopenia to several illnesses, such as cancer, type 2 diabetes, cardiovascular disease, chronic obstructive pulmonary disease, and acute and chronic critical illnesses [3,4,5,6,7]. However, there are no medications approved specifically for sarcopenia [1].

The mechanisms of sarcopenia include inflammation, oxidative stress, imbalances between protein synthesis and degradation or primary sarcopenia in healthy older adults [8,9,10]. All causes increase inflammation and reactive oxygen species (ROS) levels in the body through lipid deposition, insulin resistance, and pro-inflammatory cytokine secretion, ultimately leading to sarcopenic obesity [10,11]. Several studies have been devoted to preventing or treating sarcopenia. The accumulated evidence indicates that hypovitaminosis D is independently associated with an elevated sarcopenia risk [12]. Furthermore, dietary interventions enriched with antioxidant micronutrients—particularly vitamin A (retinol), vitamin E (α-tocopherol), selenium (Se), and oil—demonstrate efficacy in ameliorating muscle strength [13]. Although there have been reports of decreased muscle mass associated with elevated dietary inflammatory indices, the validity of antioxidant therapy as the sole treatment option for sarcopenia remains a topic of debate [14,15,16]. Moreover, resistance training and nutritional interventions, particularly high protein or essential amino acid supplementation, may be effective in improving sarcopenia. Determining the optimal duration of the intervention and the precise composition of the supplements remains a challenge [17,18,19]. The current research has confirmed that Oxidative Balance Score (OBS) is negatively correlated with the risk of sarcopenia [20,21]. However, there are gender differences in sarcopenia-related genes [22]. Consequently, a further investigation of the gender-specific association between OBS, sarcopenia, and all-cause mortality, as well as an identification of the target populations that may benefit from such an investigation, is imperative to facilitate the clinical use of OBS and enhance overall survival. The objective of this study was to evaluate the association between OBS and the risk of developing sarcopenia with low muscle mass (SLM) in gender-specific populations, utilizing data obtained from the National Health and Nutrition Examination Survey (NHANES).

## 2. Methods

### 2.1. Study Population

**Figure 1:**
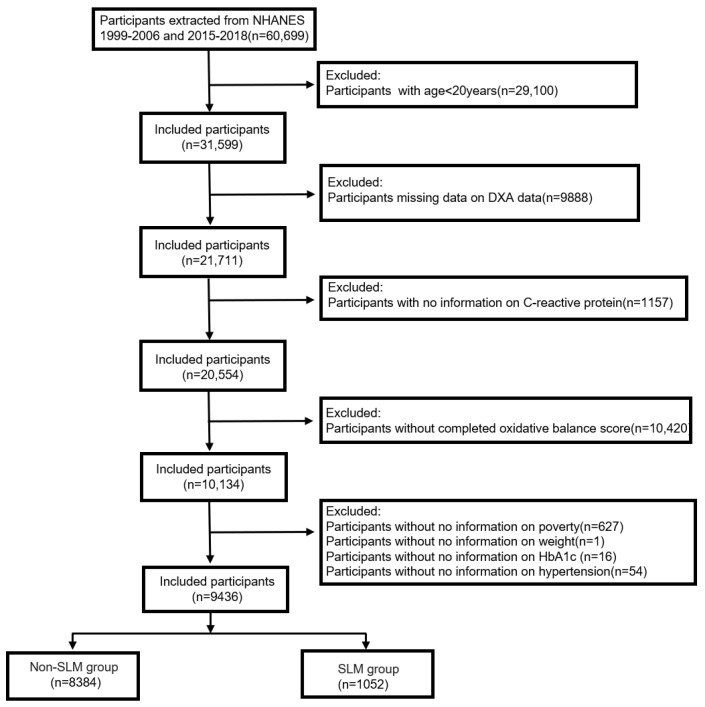
Flowchart of participant selection in this study.

The 60,699 participants in this study were drawn from representative consecutive NHANESs from 1999 to 2006 and 2015 to 2018. These cycles were selected based on the fact that the participants had filled out detailed questionnaires about their physical activity level and dietary intake, and their muscle mass was measured. The exclusion criteria included the following: (1) age under 20 (*n* = 29,100); (2) incomplete dual-energy X-ray absorptiometry (DXA) results (*n* = 9888); (3) missing c-reactive protein (CRP) data (*n* = 1157); and (4) the absence of all 20 OBS components (*n* = 10,420), and a lack of data on missing covariates such as poverty index (*n* = 627), weight (*n* = 1), glycosylated hemoglobin (HbA1c) (*n* = 16), and comorbid hypertension (*n* = 54). Ultimately, this study included 9436 participants (Figure 1).

### 2.2. SLM Measurements

Muscle mass data were accurately assessed for each participant using DXA technology (Hologic, Inc., Bedford, MA, USA). Data were collected using HologicQDR-4500 software in Apex version 3.2. Appendiceal skeletal muscle (ASM) refers to the entirety of the bone-free muscles in arms and legs. Body mass index (BMI) classifies ASM into different groups for a more comprehensive assessment of SLM. We used the National Institutes of Health (NIH)’s definition of SLM, which is as follows: ASM/BMI, male < 0.789, and female < 0.512 [23].

### 2.3. Oxidative Balance Score (Exposure)

Based on previous research on the relationship between OBS and dietary or lifestyle variables, an OBS would consist of 16 nutrients and 4 lifestyle variables [24]. The composition of dietary intake was measured by personnel with NHANES training. The first 24 h dietary review survey was conducted for all subjects and the nutrients included were magnesium, zinc, copper, selenium, dietary fiber, carotenoids, riboflavin, niacin, vitamin B6, total folate, vitamin B12, vitamin C, vitamin E, iron, and total fat. The lifestyle variables were alcohol consumption, smoking, BMI, and physical activity (PA). Among these, total fat, iron, BMI, alcohol consumption, and smoking are regarded as pro-oxidants and the remaining factors as antioxidants.

The OBSs were calculated according to the methods of Zhang et al. [25]. We used serum cotinine to express the degree of smoking. PA was measured using the Physical Activity Questionnaire (PAQ) and a participant’s weekly relative energy metabolism was calculated using the metabolic equivalent (MET). The NHANES offers recommended MET values for various exercise types. The PAQ questionnaire addresses the following activities: walking or cycling (MET = 4), work-related strenuous activity (MET = 8), moderate work-related activity (MET = 4), and vigorous physical activity during leisure time (MET = 8). PA can be calculated based on MET value, activity type, weekly frequency, and duration. We calculated PA values using the following formula: PA (MET-h/week) = MET × weekly frequency × duration of each physical activity [26].

We classified the nutrient, BMI, PA, and serum cotinine levels by gender and divided them into three groups according to their tertiles, with the antioxidants in groups 1–3 rated 0–2 and the pro-oxidants in groups 1–3 rated 2–0. The amount of alcohol consumption was divided into three categories: heavy drinkers (female ≥ 15 g/d, male ≥ 30 g/d), non-heavy drinkers (female 0–15 g/d, male 0–30 g/d), and non-drinkers with values of 0, 1, and 2. The higher the OBS, the more significant the antioxidant load.

### 2.4. Covariate Assessment

Previous studies have shown that the covariates associated with sarcopenia or OBS include sociodemographic variables, inflammatory factors, and comorbidities [9,27,28,29]. The sociodemographic variables include age, gender (male/female), ethnicity (Mexican American, non-Hispanic black, non-Hispanic white, other Hispanic, other race—including multiracial), education (less than 9th grade, 9-11th grade, High School diploma/GED, some college or associate (AA) degree, college degree or higher), and the ratio of family income to poverty level (PIR) (<1.3, 1.3–3.5, >3.5). The inflammatory factors are reflected by CRP levels and quantified by latex-assisted turbidimetry. Comorbidities consist of hypertension and diabetes. Study participants were considered smokers if they had smoked more than 100 cigarettes in their lifetime. Diabetes mellitus or hypertension that was diagnosed by a doctor and self-reported constituted the definition of disease history.

### 2.5. Statistical Analysis

We determined individual sample weights based on the NHANES recommended sample weight on the first day of the diet and obtained unbiased estimates in all analyses (1999–2002 were WTDR4YR × 2/6, 2003–2006 and 2015–2018 were WTDRD1 × 4/6). For a baseline characteristics analysis, weighted mean (standard error) was used for continuous variables and sample size (weighted percentage) was used for categorical variables. Univariate analyses of variance (ANOVA) were performed for differences in the weighted means for continuous variables, and for differences in the weighted percentages for categorical variables; the Rao–Scott χ^2^ test was performed to determine the characteristics of the entire population. R version 4.2.1 was employed for all statistical analyses (R Foundation for Statistical Computing, Vienna, Austria; http://www.r-project.org, Accessed on 5 January 2025).

## 3. Results

### 3.1. Baseline Characteristics

Participants in the SLM group had lower OBS levels (*p* < 0.001) and higher CRP levels (*p* < 0.001) when compared with the non-SLM group (Supplementary Table S1). Table 1 presents the baseline characteristics of the participants classified according to OBS. The results indicate that the average age of the study participants is 43.94 ± 0.32 years, with females comprising 49.45% of all subjects. Furthermore, there are no significant differences in age and gender among the OBS classifications. The majority of the participants are non-Hispanic whites (75.81%). An increase in OBS levels is associated with a reduction in the prevalence of SLM (*p* < 0.001). Furthermore, higher OBS levels correlate with decreased inflammatory marker CRP and metabolic marker HbA1c levels (both *p* < 0.001). Additionally, elevated OBS levels are linked to lower prevalences of diabetes and hypertension (both *p* < 0.001).

### 3.2. Association of Nutrients and Lifestyle with SLM Risk in Females/Males

**Figure 2:**
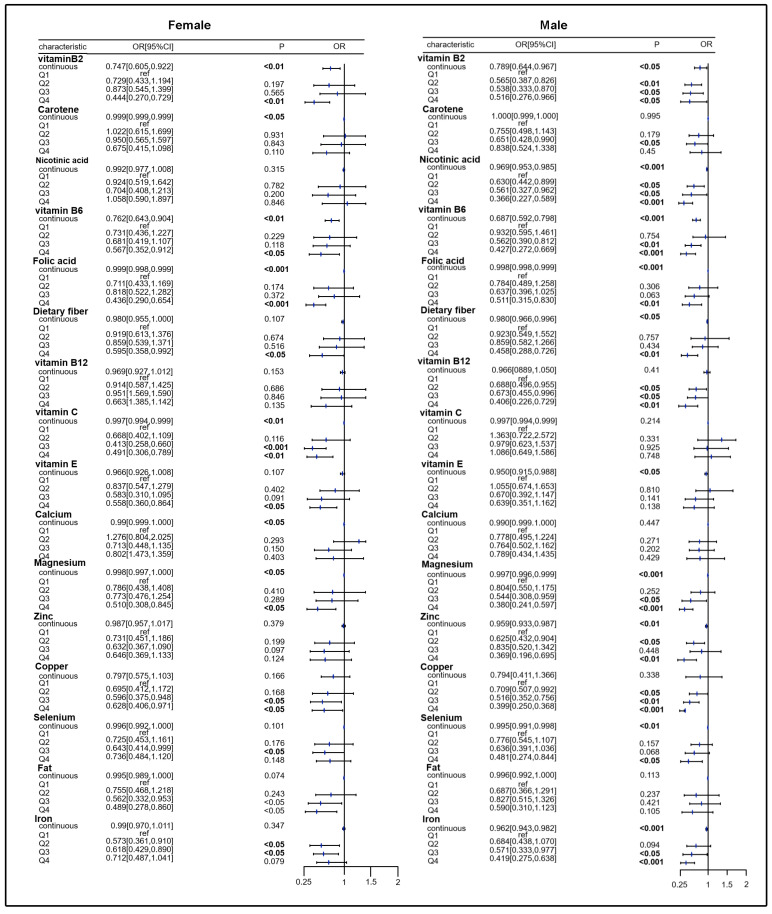
Association of individual dietary nutrients with SLM risk in females/males.

We analyzed the association between 16 nutrients and the risk of SLM under gender stratification. The results showed that the highest quartiles and continuous variables for vitamin B2, vitamin B6, folic acid, and magnesium intake were negatively associated with the risk of SLM in both males and females compared to the lowest quartile (female, *p* < 0.01, male, *p* < 0.05), and the highest quartiles and third quartiles of selenium, iron, and copper levels were negatively associated with a reduced risk of SLM in both males and females when compared with the lowest quartile (selenium, female, *p* < 0.05, male, *p* < 0.05; iron, female, *p* < 0.05, male, *p* < 0.001; and copper, female, *p* < 0.05, male, *p* < 0.001) (Figure 2). In addition, the risk of SLM was negatively associated with continuous variables for carotenoids in the female group, but in the male group, only the third quartile of carotenoid intake level was negatively associated with the risk of SLM compared to the lowest quartile of carotenoid levels (female, *p* < 0.05, male, *p* < 0.05). Additionally, the risk of SLM was negatively associated with continuous variables of vitamin E and dietary fiber intake in the male group, whereas the highest quartiles of vitamin E and dietary fiber intake were negatively associated with the risk of SLM in females when compared with the lowest quartile (vitamin E, female, *p* < 0.05, male, *p* < 0.05, dietary fiber, female, *p* < 0.05, male, *p* < 0.01) (Figure 2). Interestingly, compared to the lowest quartile, the highest quartiles of niacin, zinc, and vitamin B12 levels were only negatively associated with the risk of SLM in males, while the highest quartile of vitamin C was only negatively associated with the risk of SLM in females (niacin, female, *p* > 0.05, male, *p* < 0.001; zinc, female, *p* > 0.05, male, *p* < 0.01; vitamin B12, female, *p* > 0.05, male, *p* < 0.01; and vitamin C, female, *p* < 0.01, male, *p* > 0.05), and the continuous variable of calcium was also negatively associated with risk of SLM in females (calcium, female, *p* < 0.05) (Figure 2). Finally, continuous variables for fat were not associated with the risk of SLM in either males or females.

**Figure 3:**
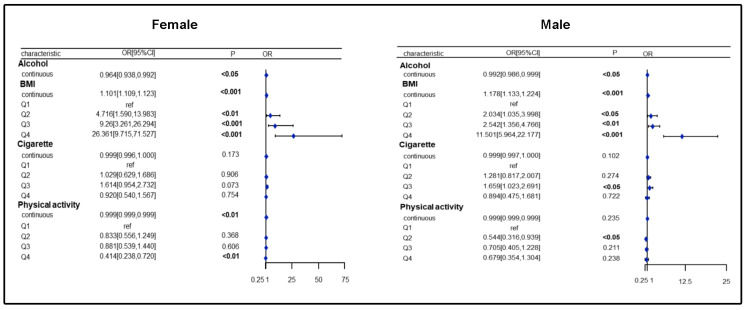
Association of lifestyle with SLM risk in females/males.

To explore the independent effects of different lifestyles on the risk of SLM by gender, an analysis was performed. After adjusting for all covariates, the result of the weighted logistic regression analysis showed that continuous variables of BMI were strongly and positively associated with the risk of SLM in the male and female groups. Compared to the lowest quartile of BMI, the highest quartile of BMI in the male and female groups led to an 11.5-fold and 26.36-fold increase in the risk of SLM, respectively (female, *p* < 0.001, male, *p* < 0.001). In addition, compared with the lowest quartile of serum cotinine levels, the third quartile of serum cotinine levels in the male group resulted in a 1.66-fold increase the risk of SLM, but not in the female group (male, *p* < 0.05). In addition, PA in the second quartile in the male group and in the highest quartile in the female group was negatively associated with the risk of SLM when compared with PA in the lowest quartile (female, *p* < 0.01, male, *p* < 0.05) (Figure 3).

### 3.3. Association of Different OBSs with SLM Risk Obtained by Gender-Stratified Analysis

Our analysis indicated that total OBS, dietary OBS, and lifestyle OBS were negatively associated with SLM risk (Supplementary Tables S2 and S3). We further analyzed the above association in the gender subgroup in Table 2. After adjusting for sociodemographic factors, CRP, HbA1c, and comorbidities, total OBS, dietary OBS, and lifestyle OBS in the highest quartile were negatively associated with the risk of SLM in both the male and female groups when compared to the lowest quartile OBSs (all *p* < 0.05). Notably, the male group had a larger OR of the highest quartile of total OBS and lifestyle OBS than the female group, while the female group had a greater OR of the various quartiles of dietary OBS. Regarding the gender–OBS interaction analysis, there was no gender interaction effect on the association between total OBS, dietary OBS, or lifestyle OBS and SLM risk (all interactions *p* > 0.05).

The specific range for the quantiles is consistent with Table 1 and Supplementary Tables S2 and S3.

The Adjusted Model is adjusted for Age, Gender, Race, Education, PIR, CRP and HbA1c, Smoke History, Hypertension, and Diabetes.

### 3.4. Restricted Cubic Spline Regression Analysis of the Nonlinear Relationship Between OBS and SLM Risk

**Figure 4:**
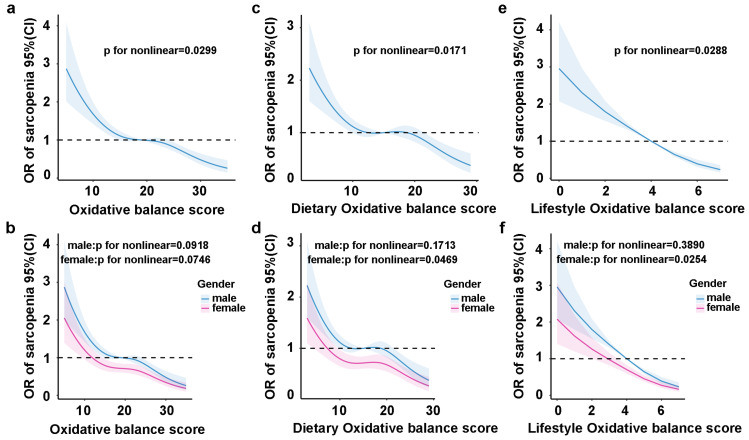
RCS regression analysis of the association between OBS and SLM risk in gender subgroups. RCS analysis revealed nonlinear correlations between total OBS and SLM risk in the total population and across different gender subgroups (**a**,**b**). Additionally, RCS analysis demonstrated nonlinear correlations between dietary OBS and SLM risk in the total population and within different gender subgroups (**c**,**d**). Finally, RCS analysis identified nonlinear correlations between lifestyle OBS and SLM risk in the total population and among gender subgroups (**e**,**f**). RCS model adjusted for age, gender, ethnicity, education, PIR, CRP, HbA1c, hypertension, smoking, and diabetes disease.

After adjusting for all covariates, we found a significant nonlinear relationship between SLM risk and total OBS in the Restricted cubic spline (RCS) regression analysis. Specifically, Figure 4a shows a decreasing SLM risk with an increasing total OBS (nonlinear *p =* 0.0299), which remains constant across gender subgroups but is not statistically significant (Figure 4b). Dietary OBS exhibited a negative nonlinear association with the risk of SLM (Figure 4c, nonlinear *p* = 0.0171), particularly within the female subgroup (Figure 4d, nonlinear *p* = 0.0469). Furthermore, lifestyle OBS also demonstrated an inverse relationship with SLM risk (Figure 4e, nonlinear *p* = 0.0288), which was similarly significant in the female subgroup (Figure 4f, nonlinear *p* = 0.0254). However, all nonlinear associations between dietary/lifestyle OBSs and SLM risk were absent in the male subgroup (Figure 4d,f, nonlinear *p* = 0.1713, nonlinear *p =* 0.3890).

### 3.5. Survival Analysis and Cox Regression Analysis of OBS and All-Cause Mortality

**Figure 5:**
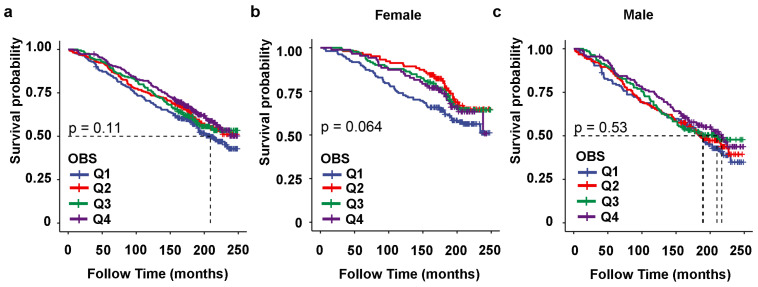
Kaplan–Meier survival analysis curves for all-cause mortality according to total OBS quartiles. Kaplan–Meier survival analysis curves for all causes mortality in the overall SLM populations (**a**), as well as in the male subgroup (**b**) and female subgroup (**c**). Total OBS: Q1 (4–12); Q2 (13–17); Q3 (18–22); and Q4 (23–35). Total OBS quartile (Q) in females: Q1 (4–13); Q2 (14–17); Q3 (18–24); and Q4 (25–32). Total OBS quartile (Q) in males: Q1 (4–11); Q2 (12–16); Q3 (17–22); and Q4 (23–35).

During a follow-up period of 168,543 months (median follow-up of 177 months), there were a total of 1049 all-cause deaths, of which 478 were in the female group and 571 were in male group. All-cause mortality is illustrated in Figure 5. Total OBS was not significantly associated with all-cause mortality among the individuals in the overall SLM population (Figure 5a, *p* = 0.11), nor in the male and female subgroups (Figure 5b,c, female, *p* = 0.064, male, *p* = 0.53).

## 4. Discussion

For the first time, our study utilized a large sample size to investigate gender differences in the association between OBS and SLM risk. The findings remained statistically significant even after adjusting for multiple confounders. Notably, females appear to derive greater benefits from antioxidant-rich diets and lifestyles.

Consistent with previous studies [20,21], our findings indicate a negative association between OBS and the risk of SLM in the general population. This negative relationship is consistent across total OBS, dietary OBS, and lifestyle-related OBS. Notably, the stratification of our study by gender revealed gender differences in how individual dietary nutrition and lifestyle factors correlate with the risk of SLM. Females demonstrated greater benefits from these associations, particularly regarding total OBS and lifestyle OBS. Furthermore, our research identified a nonlinear association between OBS and SLM risk, which was especially pronounced in females.

This study showed gender specifics in the association between 16 nutrients and SLM risk. Moreover, it was observed that females exhibited greater benefits, which is consistent with the previously reported results indicating that a high intake of B vitamins (B12, B9) and minerals (potassium, calcium, and magnesium) is strongly associated with a reduced risk of sarcopenia [30,31]. Another global study on aging and health in adults also found that increased fruit consumption was significantly associated with a reduction in the prevalence of sarcopenia and was more beneficial to females [32]. This may be because vegetables and fruits contain antioxidants such as carotenoids, selenium, and folic acid, which help counteract the ROS that damage mitochondria and worsen sarcopenia by increasing oxidative stress and impairing mitochondrial homeostasis in muscles [33]. In addition, the higher incidence of sarcopenia in females may be related to their eating habits. Postmenopausal females tend to experience lower protein and calorie intake to avoid weight gain and increased fat intake due to the fluctuations in estrogen levels in their bodies [34,35]. These behaviors can lead to a lack of multiple nutrients in females, increasing their susceptibility to sarcopenia. Although the scientific community is increasingly advocating for the use of whole foods that protect muscles rather than individual nutrients to prevent sarcopenia. Our findings suggest that it may be necessary to provide a gender-specific antioxidant diet together with the recommended quantity of dietary components such as carotene, copper, selenium, and iron to avoid excessive or inadequate intake levels.

Our analysis also showed gender differences between physical activity level and SLM risk, which may be because females tend to be more sedentary and have lower levels of activity, such as spending less time on physical activities, cycling, and gardening, compared to males [34]. Moreover, there is no positive correlation between the amount of exercise and muscle health [36]. Therefore, it is necessary to recommend the appropriate level of activity for the different genders. Furthermore, there is a reciprocal relationship between BMI and SLM. Our findings suggest that obesity is a strong risk factor for SLM, especially in females. It can lead to lipotoxicity and insulin resistance, impair fatty acid β oxidation, increase ROS production, cause mitochondrial dysfunction, and increase the secretion of pro-inflammatory cytokines. This aggravates systemic inflammation and insulin resistance in sarcopenic obesity patients, leading to muscle metabolic disorders and sarcopenia [10]. Therefore, weight loss is crucial for muscle health.

Our findings indicate that there is no significant relationship between OBS and all-cause mortality in the SLM population. However, a trend may be observed within the female subgroup, potentially due to its limited sample size. Despite previous studies demonstrating a negative association between dietary OBS and all-cause mortality, it is crucial to recognize that these studies primarily focused on diet, with limited consideration of lifestyle factors and a lack of stratification by gender [37]. Our study contributes to the resolution of this issue. Moreover, our results differ from another study that found a negative association between OBS and all-cause mortality in a population aged 55–69 years [38]. This discrepancy is likely due to the fact that our study population included people >20 years of age with SLM, so further correlation analysis by age group is warranted.

There are also some limitations to our study. First, it is difficult to determine a causal relationship between OBS and SLM because this study was a retrospective study, and this study ignored the threshold effects of antioxidants and instead assumed that all pro-oxidants and antioxidants are linearly correlated with oxidative stress. Second, this study cannot effectively reveal the effective mechanism of OBS that leads to a reduced risk of SLM, and prospective design studies are needed to demonstrate the effectiveness of OBS. Furthermore, the NHANES database used in this study may create an impression that the result applies exclusively to individuals sharing similar lifestyle patterns. Finally, the connection between drug use and oxidative stress in comorbidities has been overlooked because there is not enough information to explain the confounding factors of SLM and the associated drug use.

## 5. Conclusions

This study presents compelling evidence that OBSs are significantly and negatively correlated with the risk of SLM. Furthermore, it highlights that females appear to derive greater benefits from diets and lifestyles rich in antioxidants. This research advocates for the formulation of gender-specific antioxidant regimens, which could facilitate the development of targeted prevention strategies for individuals affected by SLM.

## Figures and Tables

**Table 1:** Baseline and characteristics of the study population.

Characteristic	Overall, N = 9436	Quartile of OBS				*p* Value
		Q1, N = 2770	Q2, N = 2470	Q3, N = 2354	Q4, N = 1842	
Age (year), Mean (S.E)	43.94 (0.32)	44.16 (0.54)	44.23 (0.51)	44.22 (0.51)	43.06 (0.46)	0.15
Gender, *n* (%)						>0.9
Female	4532 (49.45)	1336 (50.29)	1182 (49.07)	1144 (49.20)	870 (49.21)	
Male	4904 (50.55)	1434 (49.71)	1288 (50.93)	1210 (50.80)	972 (50.79)	
Race, *n* (%)						<0.001
Non-Hispanic White	5209 (75.81)	1322 (69.21)	1300 (72.14)	1418 (80.07)	1169 (82.15)	
Non-Hispanic Black	1769 (9.66)	744 (15.31)	471 (10.86)	327 (6.70)	227 (5.57)	
Mexican American	1748 (6.12)	489 (5.87)	497 (7.07)	429 (5.65)	333 (5.93)	
Other Hispanic	368 (3.32)	112 (3.39)	108 (4.55)	93 (2.90)	55 (2.38)	
Other/multiracial	342 (5.09)	103 (6.22)	94 (5.37)	87 (4.70)	58 (3.98)	
Education, *n* (%)						<0.001
Less Than 9th Grade	902 (3.80)	381 (6.72)	255 (4.16)	180 (2.79)	86 (1.37)	
9–11th Grade	1223 (8.39)	459 (11.05)	321 (9.25)	277 (7.44)	166 (5.60)	
High School Grad/GED	2220 (23.71)	707 (27.33)	619 (25.46)	533 (24.13)	361 (17.34)	
Some College or AA degree	2772 (32.34)	810 (34.84)	723 (32.44)	717 (32.06)	522 (29.77)	
College Graduate or above	2311 (31.76)	412 (20.05)	548 (28.68)	646 (33.58)	705 (45.92)	
PIR, *n* (%)						<0.001
<1.3	2110 (15.36)	792 (21.80)	571 (15.82)	442 (12.51)	305 (11.06)	
>3.5	3767 (50.52)	851 (40.01)	947 (49.64)	1,039 (53.57)	930 (59.56)	
1.3–3.5	3559 (34.12)	1127 (38.19)	952 (34.54)	873 (33.93)	607 (29.38)	
CRP, Mean (S.E)	0.40 (0.01)	0.50 (0.03)	0.38 (0.02)	0.39 (0.02)	0.33 (0.03)	<0.001
HbA1c (%)	5.40 (0.01)	5.51 (0.04)	5.42 (0.03)	5.37 (0.02)	5.30 (0.03)	<0.001
SLM, *n* (%)						<0.001
Yes	1052 (7.25)	417 (11.32)	307 (8.36)	225 (5.81)	103 (3.21)	
No	8483 (92.75)	2353 (88.68)	2163 (91.64)	2129 (94.19)	1739 (96.79)	
Smoke, *n* (%)						<0.001
Yes	4502 (47.69)	1499 (55.03)	1201 (47.69)	1082 (45.54)	720 (42.06)	
No	4934 (52.31)	1271 (44.97)	1269 (52.31)	1272 (54.46)	1122 (57.94)	
Hypertension, *n* (%)						<0.001
Yes	2736 (26.29)	954 (31.62)	706 (25.14)	656 (26.02)	420 (21.96)	
No	6700 (73.71)	1816 (68.38)	1764 (74.86)	1698 (73.98)	1422 (78.04)	
Diabetes mellitus, *n* (%)						0.002
Yes	914 (6.93)	332 (9.29)	259 (7.14)	198 (6.31)	125 (4.82)	
No	8522 (93.07)	2438 (90.71)	2211 (92.86)	2156 (93.69)	1717 (95.18)	

**Table 2:** The association between the different OBSs and SLM risk by gender.

	Q1	Q2 (OR 95%CI)	*p*	Q3 (OR 95%CI)	*p*	Q4 (OR 95%CI)	*p*	*p* for Trend	*p* for Interaction
Total OBS									
Unadjusted									
Female	Ref	0.97 (0.64, 1.46)	0.882	0.67 (0.43, 1.05)	0.082	0.31 (0.17, 0.55)	<0.001	<0.001	0.015
Male	Ref	0.55 (0.38, 0.82)	0.003	0.36 (0.23, 0.57)	<0.001	0.23 (0.13, 0.39)	<0.001	<0.001	
Adjusted									
Female	Ref	0.94 (0.54, 1.62)	0.808	0.71 (0.42, 1.21)	0.202	0.34 (0.19, 0.61)	0.003	0.001	0.219
Male	Ref	0.59 (0.40, 0.86)	0.007	0.43 (0.26, 0.72)	0.002	0.35 (0.19, 0.65)	0.001	<0.001	
Dietary OBS									
Unadjusted									
Female	Ref	1.05 (0.68, 1.63)	0.827	0.75 (0.49, 1.15)	0.189	0.50 (0.31, 0.81)	0.006	0.006	0.008
Male	Ref	0.53 (0.36, 0.77)	0.001	0.47 (0.30, 0.74)	0.002	0.25 (0.15, 0.42)	<0.001	<0.001	
Adjusted									
Female	Ref	0.95 (0.56, 1.59)	0.833	0.78 (0.47, 1.29)	0.319	0.54 (0.34,0.86)	0.010	0.018	0.159
Male	Ref	0.58 (0.40, 0.86)	0.007	0.56 (0.34, 0.91)	0.022	0.41 (0.24, 0.70)	0.002	0.001	
Lifestyle OBS									
Unadjusted									
Female	Ref	0.66 (0.47, 0.93)	0.018	0.53 (0.37, 0.76)	<0.001	0.13 (0.07, 0.26)	<0.001	<0.001	0.941
Male	Ref	0.57 (0.36, 0.90)	0.017	0.51 (0.32, 0.81)	0.005	0.29 (0.15, 0.56)	<0.001	<0.001	
Adjusted									
Female	Ref	0.60 (0.42, 0.86)	0.007	0.45 (0.32, 0.63)	<0.001	0.14 (0.07, 0.29)	<0.001	<0.001	0.795
Male	Ref	0.56 (0.34, 0.92)	0.023	0.45 (0.28, 0.72)	0.001	0.25 (0.12, 0.51)	<0.001	<0.001	

## Data Availability

Data used for this study are available on the NHANES website: https://wwwn.cdc.gov/nchs/nhanes/. Accessed on 5 January 2025.

## Supplementary Materials

The following supporting information can be downloaded at https://www.mdpi.com/article/10.3390/healthcare13080910/s1, Table S1: Baseline and characteristics of the study population; Table S2: Logistic regression analysis models showing the associations between total OBS and SLM.; and Table S3: OR estimates for associations between dietary/lifestyle OBS and SLM.
