# Supplementary material for: Antioxidant Diets and Lifestyles Could Mitigate the Risk of Sarcopenia with Low Muscle Mass in Women: A Retrospective Study
Source: Healthcare (Basel). 2025 Apr 15;13(8):910. doi: 10.3390/healthcare13080910 (PMC12026764; doi:10.3390/healthcare13080910)
Supplement: Supplementary file 1 [file healthcare-13-00910-s001.zip › healthcare-3444119-supplementary.pdf]

Antioxidant Diets and Lifestyles Could Mitigate the Risk of Sarcopenia with Low Muscle Mass in Women: A Retrospective Study

Shanshan Li [1,2#], Yiqiong Zhang[1,2#], Qian Li[1,2], Wenjun Liu[1*], Yue Wu[1,2,3*]

Supplementary Table S1. Baseline and characteristics of the study population.

Characteristic	Overall, N = 9436	Non-SLM, N = 8384	SLM, N = 1052	*P* Value
Age (year), Mean (S.E)	43.94 (0.32)	43.02 (0.35)	55.69 (0.83)	<0.001
Gender, n (%)				0.200
female	4,532 (49.45)	4,052 (49.76)	480 (45.46)	
male	4,904 (50.55)	4,332 (50.24)	572 (54.54)	
Race, n (%)				<0.001
Mexican American	1,748 (6.12)	1,345 (5.50)	403 (14.06)	
Other Hispanic	368 (3.32)	317 (3.01)	51 (7.26)	
Non-Hispanic White	5,209 (75.81)	4,686 (76.30)	523 (69.51)	
Non-Hispanic Black	1,769 (9.66)	1,731 (10.18)	38 (2.99)	
Other/multiracial	342 (5.09)	305 (5.00)	37 (6.18)	
Education, n (%)				<0.001
Less Than 9th Grade	902 (3.80)	622 (3.00)	280 (14.16)	
9-11th Grade	1,223 (8.39)	1,059 (7.94)	164 (14.07)	
High School Grad/GED	2,220 (23.71)	1,959 (23.12)	261 (31.21)	
Some College or AA degree	2,772 (32.34)	2,557 (33.04)	215 (23.39)	
College Graduate or above	2,311 (31.76)	2,180 (32.90)	131 (17.17)	
PIR, n (%)				<0.001
<1.3	2,110 (15.36)	1,777 (14.91)	333 (21.13)	
>3.5	3,767 (50.52)	3,506 (51.72)	261 (35.20)	
1.3-3.5	3,559 (34.12)	3,101 (33.37)	458 (43.66)	
Total OBS, Mean (S.E)	20.16 (0.19)	20.40 (0.20)	17.07 (0.42)	<0.001
CRP, Mean (S.E)	0.40 (0.01)	0.38 (0.01)	0.61 (0.04)	<0.001
HbA1c, Mean (S.E)	5.40 (0.12)	5.36 (0.02)	6.00 (0.10)	<0.001
Smoke, n (%)				0.7

Yes	4,502 (47.69)	3,976 (47.60)	526 (48.84)	
No	4,934 (52.31)	4,408 (52.40)	526 (51.16)	
hypertension, n (%)				<0.001
Yes	2,736 (26.29)	2,236 (24.49)	500 (49.33)	
No	6,700 (73.71)	6,148 (75.51)	552 (50.67)	
Diabetes mellitus, n (%)				<0.001
Yes	914 (6.93)	679 (5.69)	235 (22.75)	
No	8,522 (93.07)	7,705 (94.31)	817 (77.25)	

The specific range for the OBS quartile (Q): Q1(3-14); Q2(15-20); Q3(21-26); Q4(27-37).

Supplementary Table S2. Logistic regression analysis models showing the associations between total OBS and SLM.

Total OBS	Crude model		Model1		Model2		Model3	
	OR 95%CI	P value	OR 95%CI	P value	OR 95%CI	P value	OR 95%CI	P value
Q1	Ref		Ref		Ref		Ref	
Q2	0.72(0.53, 0.97)	0.031	0.71(0.50, 0.99)	0.043	0.73(0.52, 1.03)	0.072	0.73(0.52,1.03)	0.076
Q3	0.48(0.35, 0.68)	<0.001	0.52(0.35, 0.78)	0.002	0.54(0.36, 0.80)	0.003	0.54(0.36, 0.80)	0.003
Q4	0.26(0.17, 0.39)	<0.001	0.32(0.21, 0.50)	<0.001	0.35(0.23, 0.53)	<0.001	0.35(0.22, 0.54)	<0.001
P for trend		<0.001		<0.001		<0.001		<0.001

Model 1: Adjusted for age, sex, race, education, and poverty-income ratio.

Model 2: Additionally adjusted for CRP and HbA1c.

Model 3: Additionally, adjusted for smoke, hypertension and diabetes.

The specific range for the quantiles is consistent with Table 1.

Crude model: Unadjusted model.

Supplementary Table S3. OR estimates for associations between dietary/lifestyle OBS and SLM.

OBS	Q1	Q2 (OR 95%CI)	*P*	Q3 (OR 95%CI)	*P*	Q4 (OR 95%CI)	*P*	*P* for trend
Dietary OBS								
Crude model	Ref	0.73(0.54, 0.98)	0.037	0.58(0.42, 0.80)	0.001	0.35(0.24, 0.50)	<0.001	<0.001
Model 1	Ref	0.72(0.52, 1.00)	0.050	0.64(0.44, 0.93)	0.019	0.45(0.31, 0.64)	<0.001	<0.001
Model 2	Ref	0.73(0.52,1.03)	0.072	0.64(0.44, 0.94)	0.023	0.47(0.32, 0.67)	<0.001	<0.001
Model 3	Ref	0.73(0.51, 1.02)	0.068	0.64(0.44, 0.93)	0.022	0.46(0.32, 0.68)	<0.001	<0.001
Lifestyle OBS								
Crude model	Ref	0.61(0.44, 0.85)	0.005	0.52(0.37, 0.71)	<0.001	0.20(0.12, 0.35)	<0.001	<0.001
Model 1	Ref	0.57(0.41, 0.78)	<0.001	0.45(0.33, 0.62)	<0.001	0.19(0.11, 0.32)	<0.001	<0.001
Model 2	Ref	0.60(0.44, 0.83)	0.002	0.46(0.34, 0.63)	<0.001	0.21(0.12, 0.35)	<0.001	<0.001
Model 3	Ref	0.58(0.42, 0.80)	0.001	0.45(0.33, 0.61)	<0.001	0.20(0.12, 0.34)	<0.001	<0.001

Model 1: Adjusted for age, sex, race, education, and poverty-income ratio.

Model 2: Additionally adjusted for CRP and HbA1c.

Model 3: Additionally, adjusted for smoke, hypertension and diabetes.

The specific range for the quantiles: Dietary OBS: Q1 [1,10]; Q2[11,15]; Q3[16,21]; Q4 [22,30];

Lifestyle OBS: Q1 [0,3]; Q2 [4,4]; Q3 [5,5]; Q4 [6,7].

Crude model: Unadjusted model
